# Supplementary material for: Chromothripsis during telomere crisis is independent of NHEJ, and consistent with a replicative origin
Source: Genome Res. 2019 May;29(5):737–49. doi: 10.1101/gr.240705.118 (PMC6499312; doi:10.1101/gr.240705.118)
Supplement: Supplemental Material [file supp_gr.240705.118_Supplemental_file_1.zip › contigs/annotated_contigs/DB115/contig.2.DB115_length_555_mean_cov_9.18918918919.docx]

**DB115_length_555_mean_cov_9.18918918919**

```
TGATAGTTCCAGTTCATCCATGTTCTCATAAGCACTCTGTATGAACAGTCTTTTTAAGTTTAGCCATTCTCATCAGTGTGCAGTCATAT
 >chr13:27190370-27190623 + E=1e-140
CTCCTGCGGTTTTAATTTGCCTCTCCTTAATGACTAATGCTGTTGAGCATTTTTCATGTGCTTGCTTGCCATCTGCATAACTTCTTTAG

TGAAGTGTCTGTTCAAATCTTGTGCCCATTTTAAGAAATTGGGTACTTTATTATAGATTTTCAAGAATTCTTTA|T|GTGATTCAATTA
                                                                           >chr13:27192
AACCTCTTTCCCTTCTAAATTACCCAGTCTTGGGAGTATCTTTTTAGCAGTGTGAAAACAGACGAATATGAAGTCTTAGCCATTAATAG
151-27192454 + E=1e-170
TGAGTTCACCCATTAACACTTACTGTGATTATTTGGAAGGCCATAGTCACTATTAGTGCCTCCTTTTTGTTAAAAAAATTAATACTTTC

TTGTCCATTGCTGACTTTATTACAATTTTTTCCTGTAATGGTTTGTTATTTATATATCCAGTATCTTCTAGTTGTTTGTACGAAATCAA

AATATTGATCATGCAGATAACAC
```
